# Variational Quantum Process Tomography of Non-Unitaries

**DOI:** 10.3390/e25010090

**Published:** 2023-01-01

**Authors:** Shichuan Xue, Yizhi Wang, Yong Liu, Weixu Shi, Junjie Wu

**Affiliations:** Institute for Quantum Information & State Key Laboratory of High Performance Computing, College of Computer Science and Technology, National University of Defense Technology, Changsha 410073, China

**Keywords:** quantum process tomography, variational quantum algorithm, non-unitary quantum process

## Abstract

Quantum process tomography is a fundamental and critical benchmarking and certification tool that is capable of fully characterizing an unknown quantum process. Standard quantum process tomography suffers from an exponentially scaling number of measurements and complicated data post-processing due to the curse of dimensionality. On the other hand, non-unitary operators are more realistic cases. In this work, we put forward a variational quantum process tomography method based on the supervised quantum machine learning framework. It approximates the unknown non-unitary quantum process utilizing a relatively shallow depth parametric quantum circuit and fewer input states. Numerically, we verified our method by reconstructing the non-unitary quantum mappings up to eight qubits in two cases: the weighted sum of the randomly generated quantum circuits and the imaginary time evolution of the Heisenberg *XXZ* spin chain Hamiltonian. Results show that those quantum processes could be reconstructed with high fidelities (>99%) and shallow depth parametric quantum circuits (d≤8), while the number of input states required is at least two orders of magnitude less than the demands of the standard quantum process tomography. Our work shows the potential of the variational quantum process tomography method in characterizing non-unitary operators.

## 1. Introduction

Quantum process tomography is a fundamental and indispensable technique in quantum information processing [1]. Recently, it has been increasingly crucial in benchmarking and verifying the performance of a quantum device and its dynamics when the system goes larger.

However, standard quantum process tomography (SQPT) [2,3] is cursed by the exponentially exploding Hilbert space dimension used to represent the quantum process, which needs to prepare an informationally complete set of input states and perform the standard quantum state tomography on the corresponding output quantum states [3,4,5]. Specifically, it requires $4^{n}$ input states and $4^{2n}$ quantum measurements for an *n*-qubit quantum process. Such exponential overhead severely constrains the problem size on which SQPT can be feasibly conducted. Currently, SQPT has only been experimentally implemented up to three qubits [6,7,8,9,10,11]. Many alternative proposals are put forward to address these problems, including compressed sensing tomography [12,13] and ansatz-based tomography [14,15], but at the cost of assuming specific structures of the unknown quantum process. While other benchmarking protocols only partially characterize a quantum system, including randomized benchmarking [16,17], direct fidelity estimation [18], and so on. Therefore, quantum process tomography still plays an indispensable role since it is a comprehensive characterization of quantum systems with full information and no prior assumptions. On the other hand, realistic quantum processes are often non-unitary operators. So characterizing a non-unitary quantum process is a practical problem.

Recently, quantum machine learning combined with parametric quantum circuit (PQC) has been making considerable progress [19,20,21,22,23,24]. Reference [25] showed that a gradient-based quantum machine learning algorithm could efficiently extract the information of certain quantum states encoded in a PQC, after which the unknown quantum state can be reconstructed classically with high fidelity using the optimal parameters of the PQC. Further in Ref. [26], PQC with relatively shallow depth demonstrated the capability of characterizing unitary mappings with fewer input states.

**Figure 1:**
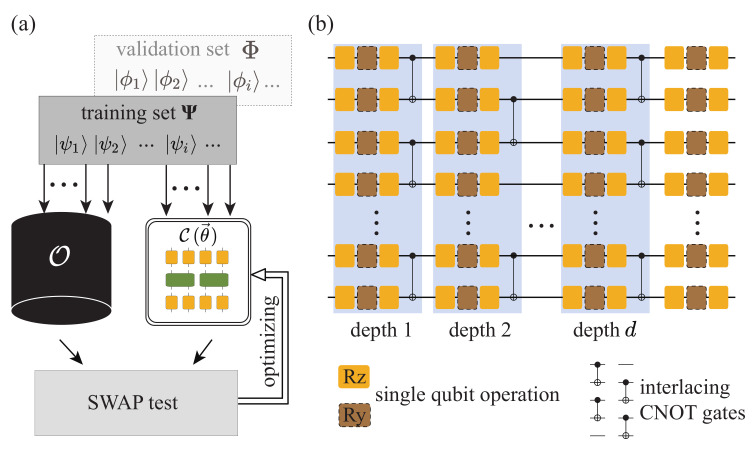
(**a**) The general framework of our VQPT method. By training the PQC with the quantum states in the training set Ψ and validation set Φ and optimizing the parameters in $C(\vec{\theta })$ based on a gradient-descent approach, the PQC $C(\vec{\theta })$ gradually approximates the physical quantum process O. (**b**) The structure of the PQC. It begins and ends with a single-qubit layer. Each two-qubit layer is counted as a depth, and the circuit contains *d* depths of operations and ends with a single-qubit layer.

In this work, we propose a variational quantum process tomography (VQPT) method of non-unitaries based on the supervised quantum machine learning framework, extending the work in Ref. [26]. As shown in Figure 1a, we utilize a *d*-depth PQC $C(\vec{\theta })$ to approximate the unknown quantum process denoted by O, where $\vec{\theta }$ is the parameter list encoding the information to be optimized in this PQC. As shown in Ref. [26], when O is a unitary, we randomly prepare a training set of *N* quantum states $|\psi_{j}\rangle$ to train the PQC, each of which is separately fed into the unknown quantum process O and the PQC $C(\vec{\theta })$. Regarding non-unitary cases, we put forward two methods in this paper—(1) superposing unitary PQCs to approximate the non-unitary; (2) transforming the non-unitary problem into a unitary one—to tackle the problem. Then, similarly, as long as each pair of output quantum states, $O|\psi_{j}\rangle$ and $C(\vec{\theta })|\psi_{j}\rangle$ are identical, and that *N* is large enough, the unknown quantum process should be approximated by $C(\vec{\theta })$ no matter O is unitary or not. As a result, all the information of O is stored in the parameters $\vec{\theta }$, and we can systematically reconstruct O from those parameters using a classical computer. We numerically verified our approach by reconstructing two typical non-unitary processes: the weighted sum of the randomly generated quantum circuits and the imaginary time evolution of the Heisenberg *XXZ* spin chain Hamiltonian, respectively. Numerical results show that we could reconstruct a non-unitary quantum process up to 8 qubits with an average gate fidelity higher than 99%, and the number of required input quantum states is smaller than that required by SQPT by at least 2 orders of magnitude.

Compared to other tomography approaches, our method has several advantages. First, combined with variational optimization, it avoids exponential measurements and complicated quantum state tomography subroutines in SQPT. It only requires measuring a single qubit for each configuration, hence is less prone to errors with relatively shallow circuit and simple measurement [27,28]. Second, with a small number of input states, our approach greatly reduces the exponential state preparation overheads and reconstructs O with high fidelities. And last, numerical simulations demonstrate the capability of our method on non-unitary quantum processes up to eight-qubit cases, further proving its generalities and potentials.

This paper is organized as follows. In Seciton Section 2, we introduce the scheme of our VQPT for non-unitary processes. In Secitons Section 3 and Section 4, we give two cases for solving the non-unitary quantum process tomography. We verified our method with numerical simulations of non-unitary quantum process tomography for the weighted sum of the randomly generated quantum circuit in Seciton Section 3 and the imaginary time evolution of a Heisenberg *XXZ* spin chain Hamiltonian in Seciton Section 4. We conclude in Section 5.

## 2. Variational Algorithm for Approximating Non-Unitary Quantum Processes with Parametric Quantum Circuits

**Figure 2:**
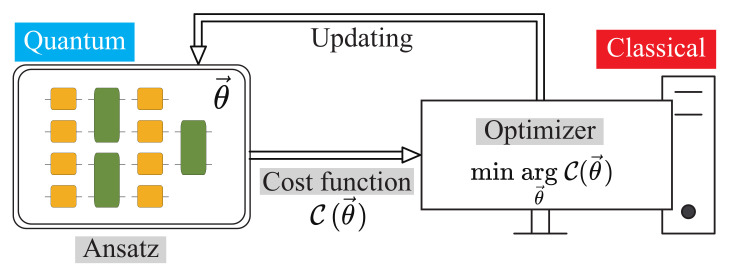
Framework of the variational quantum algorithm consisting of the ansatz, cost function and optimizer.

There are three main components in variational quantum algorithms: ansatzes, cost function, and optimization methods. Figure 2 gives an illustrative framework of variational quantum algorithms (VQA) [29]. It is a quantum-classical hybrid architecture. In this section, we describe the variational algorithm for non-unitary quantum processes from the above three aspects.

### 2.1. Ansatz of the Parametric Quantum Circuit

Ansatz is an essential aspect of a VQA. It encodes the parameters $\vec{\theta }$, and can be further trained to minimize the cost function. The specific structure of an ansatz varies from each other depending on the task. Typical ansatzes include hardware-efficient ansatz [30], variational Hamiltonian ansatz [31], and so on.

Here in our work, the design of our PQC is shown in Figure 1b, where interlaced layers of single-qubit gates and two-qubits CNOT gates are used. Such an organization facilitates the quick generation of entanglement between qubits, thus making it possible to approximate complicated quantum processes. Specifically, each two-qubit layer is counted as a depth, varying between odd and even depth. Each single-qubit layer contains three rotational gates ($R_{z}$, $R_{y}$, and $R_{z}$) on each qubit, where $R_{y}$ and $R_{z}$ are defined as

(1) $$\begin{array}{cc} R_{y}\left(\theta \right)= & \left[\begin{array}{cc} \cos\frac{\theta }{2} & -\sin\frac{\theta }{2}\;\\ \sin\frac{\theta }{2} & \cos\frac{\theta }{2} \end{array}\right];\\ R_{z}\left(\theta \right)= & \left[\begin{array}{cc} e^{-i\frac{\theta }{2}} & 0\\ 0 & e^{i\frac{\theta }{2}} \end{array}\right]. \end{array}$$

The sequences $R_{z}$, $R_{y}$, and $R_{z}$ ensure that arbitrary single qubit rotations can be realized with appropriate parameters. Our PQC ends with a single-qubit layer. As a result, the total number of parameters for such a circuit with *n* qubits and *d* depths is 3n(d+1).

It is noted that in practice, the design of the PQC should consider the underlying quantum hardware, especially the choice and pattern of the two-qubit gates. Hence the ansatz is not fixed, and our method is a generalized framework with various circuit ansatzes.

### 2.2. Cost Function on the Training Set

In our method, we build a cost function on the training set, which evaluates the distance between $C(\vec{\theta })$ and the target O. Concretely, we first randomly generate a training set of *N* quantum states, denoted as $\Psi =\left\{|\psi_{1}\rangle ,|\psi_{2}\rangle ,\ldots ,|\psi_{N}\rangle \right\}$. Here, a random quantum state is generated by applying an $R_{y}$ gate with random parameters onto each qubit and controlled phase (CZ) gates with random control and target qubits. Each state $|\psi_{j}\rangle$ is fed into the unknown quantum process O and the PQC $C(\vec{\theta })$, with the output quantum states denoted as $|\psi_{j}^{\mathrm{ideal}}\rangle =O|\psi_{j}\rangle$ and $|\psi_{j}^{\mathrm{out}}\rangle =C(\vec{\theta })|\psi_{j}\rangle$. Then, we compute the Euclidean distance between $|\psi_{j}^{\mathrm{ideal}}\rangle$ and $|\psi_{j}^{\mathrm{out}}\rangle$, which is

(2) |ψjout〉−|ψjideal〉2=Re〈ψjideal|ψjideal〉+〈ψjout|ψjout〉−2〈ψjideal|ψjout〉.

The inner product on the right-hand side of Equation (2) can be efficiently computed with a quantum computer using a generalized SWAP-test algorithm [26]. Then, the cost function $f(\vec{\theta })$ is defined as the summation of the distance obtained over all input states, which is

(3) f(θ→)=1N∑j=1N|ψjout〉−|ψjideal〉2=1N∑j=1NRe〈ψjideal|ψjideal〉+〈ψjout|ψjout〉−2〈ψjideal|ψjout〉,

namely, $f(\vec{\theta })$ is the mean square error between the two sets of output quantum states.

### 2.3. Gradient-Based Optimization

The cost function $f(\vec{\theta })$ is a hybrid quantum-classical function, and its gradient can be estimated based on the chain rule, where it contains functions to be evaluated with a quantum computer and functions to be evaluated on a classical computer:

(4) $$\begin{array}{c} \frac{\partial f(\vec{\theta })}{\partial {\vec{\theta }}_{j}}=\frac{\partial f(\vec{\theta })}{\partial S(\vec{\theta })}\frac{\partial S(\vec{\theta })}{\partial {\vec{\theta }}_{j}}=\frac{\partial f(\vec{\theta })}{\partial S(\vec{\theta })}\left(\frac{1}{2}S\left({\vec{\theta }}_{j}^{+}\right)-\frac{1}{2}S\left({\vec{\theta }}_{j}^{-}\right)\right). \end{array}$$

S in our case means the generalized SWAP-test circuit with parametric quantum gates, and $\partial S\left(\vec{\theta }\right)/\partial {\vec{\theta }}_{j}$ can be computed using the parameter-shift rule [32], where ${\vec{\theta }}_{j}$ denotes the *j*-th parameter in the parameter list $\vec{\theta }$, and ${\vec{\theta }}_{j}^{\pm }={\vec{\theta }}_{j}\pm \frac{\pi }{2}$. Hence, the gradient of the cost function $f(\vec{\theta })$ can be computed following Ref. [33], which proposed a method to embed Equation (4) into a classical automatic differentiation framework, such that the gradient of the hybrid quantum-classical cost function can be automatically computed using a hybrid quantum-classical computer. The gradient can then be fed into a gradient-based optimizer to minimize the cost function $f(\vec{\theta })$.

### 2.4. Evaluation Criteria

After the training, we evaluate experimental results between O and $C(\vec{\theta })$ using the average gate fidelity [34]. The average gate fidelity $F_{\mathrm{avg}}$ between the rebuilt $C(\vec{\theta })$ and the actual O is given by

(5) $$\begin{array}{c} F_{\mathrm{avg}}\left(C\left(\vec{\theta }\right),O\right)=\int d\psi \langle \psi \left|C{\left(\vec{\theta }\right)}^{†}O(|\psi \rangle \langle \psi |)C\left(\vec{\theta }\right)\right|\psi \rangle . \end{array}$$

In addition, we borrow the idea from supervised machine learning and design a validation set to test the generalization ability of the training outcomes. Since during training, we do not know whether our PQC is expressive enough to represent O, and whether the number of input states is enough to ensure convergence to O. Moreover, in practice, we may also have the problem of overfitting such that the optimal $C(\vec{\theta })$ is very distinct from O, but the cost function $f(\vec{\theta })$ has already converged to 0. To overcome these problems, we generate another set of *N* input states denoted as $\Phi =\left\{|\phi_{1}\rangle ,|\phi_{2}\rangle ,\ldots ,|\phi_{N}\rangle \right\}$, i.e., the validation set, which is independent of the training set. After the training process, we feed each $|\phi_{j}\rangle$ into the unknown quantum process and the resulting optimal PQC, obtaining two outputs $|\phi_{j}^{\mathrm{ideal}}\rangle =O|\phi_{j}\rangle$ and $|\phi_{j}^{\mathrm{out}}\rangle =C(\vec{\theta })|\phi_{j}\rangle$. Then, we compute the quantum fidelity between $|\phi_{j}^{\mathrm{ideal}}\rangle$ and $|\phi_{j}^{\mathrm{out}}\rangle$ efficiently through the SWAP-test [35] on a quantum computer and summarize all the instances of the validation set, which is defined as the accuracy

(6) accuracy(C(θ→),O)=1N∑j∈ΦNRe〈ϕjideal|ϕjout〉.

It is noted that accuracy is a faithful tool. If accuracy is close to one, the PQC $C(\vec{\theta })$ we obtained can be well generalized to the new input validation states. The values of accuracy and $F_{\mathrm{avg}}$ are indeed strongly correlated in our numerical simulation. Therefore, we can pick out the simulation with a larger accuracy value as a more faithful reconstruction of O. Moreover, the accuracy is an efficient evaluation criterion. Since the direct characterization of the distance between O and $C(\vec{\theta })$ as required in Equation (5) scales exponentially with the number of qubits *n*, it is possible to determine whether the training is successful or not based on the accuracy, without resorting to the complete characterization of $F_{\mathrm{avg}}$.

## 3. Case Study I: Weighted Sum of the Randomly Generated Quantum Circuits

For an *n*-qubit randomly generated quantum circuit (RQC) [36], we first apply Hadamard gates to initialize the state to a symmetric superposition. Then, the circuit is organized by depth, including CZ gates alternating between odd and even configurations to entangle neighboring qubits and randomly chosen single-qubit gate (*T*, $\sqrt{X}$ or $\sqrt{Y}$). Finally, Hadamard gates are applied to each qubit. It is noted that such randomly generated quantum circuits are hard for efficient simulation on a classical computer [36,37].

Here, we take the weighted sum of RQC as the target non-unitary operator O

(7) $$\begin{array}{c} O=pI+\left(1-p\right)U_{\mathrm{RQC}}, \end{array}$$

where the parameter *p* denotes the weighted probability that the operator is an identity matrix.

### 3.1. Methods

**Figure 3:**
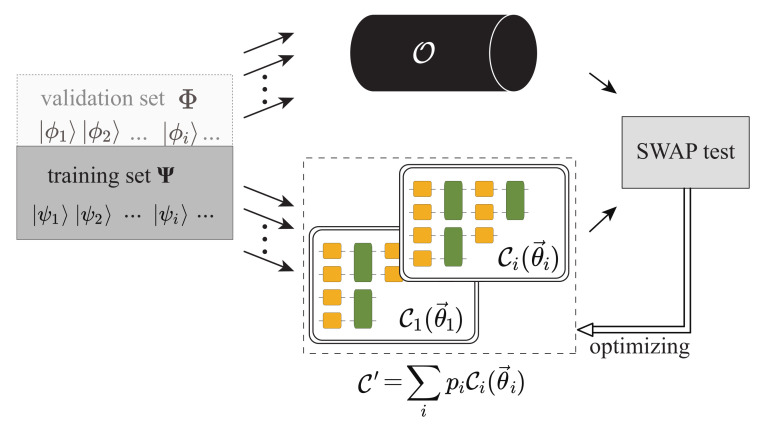
Utilizing weighted sum of unitary PQCs to approximate the non-unitary operator.

For the non-unitary operator in Equation (7), we correspondingly utilize the weighted sum of the unitary PQCs to approximate the target non-unitary quantum process

(8) $$\begin{array}{c} C^{\prime }\left({\vec{\theta }}_{i},p_{i}\right)=\sum_{i}p_{i}C_{i}\left({\vec{\theta }}_{i}\right),\sum p_{i}=1,\; \end{array}$$

where $p_{i}$ are the weighting parameters and $C_{i}\left({\vec{\theta }}_{i}\right)$ are the corresponding individual unitary PQCs as shown in Figure 3. Here in the known structure case, we assume that $C^{\prime }\left(\vec{\theta },p\right)=pI+\left(1-p\right)C\left(\vec{\theta }\right).$

When dealing with unitary mappings, the cost function in Equation (3) merely involves the third term with variational parameters since $|\psi_{j}^{\mathrm{ideal}}\rangle$ and $|\psi_{j}^{\mathrm{out}}\rangle$ are both norm-1 state vectors. In terms of such non-unitary cases, these two output states cannot be kept at norm one, depending on the circuit operations $C^{\prime }$. Therefore, the cost function involves both circuit parameters ${\vec{\theta }}_{i}$ and weighting parameters $p_{i}$:

(9) f(θ→i,pi)=1N∑j=1NRe〈ψjideal|ψjideal〉+〈ψjout|ψjout〉−2〈ψjideal|ψjout〉=1N∑j=1NRe〈ψjideal|ψjideal〉+〈ψj|C′†C′|ψj〉−2〈ψjideal|C′|ψj〉,

Hence, the gradient calculation and consequent optimization are related to the collaborative optimization of both parameters ${\vec{\theta }}_{i}$ and $p_{i}$. In Section 3.2, we give concrete examples of the non-unitary quantum process and the specific form of cost function and its gradient.

### 3.2. Numerical Results

- Fixed weighted sum of PQCs

**Figure 4:**
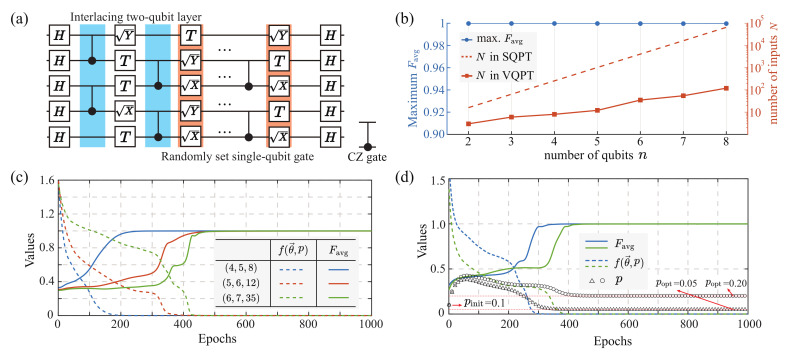
Numerical results on the weighted sum of the RQCs utilizing the weighting PQCs. (**a**) A typical five-qubit RQC organization with interlacing two-qubit layer and randomly set single-qubit gate. (**b**) Scalability tests from two-qubit to eight-qubit quantum processes. The left-axis (blue circle) shows the maximum $F_{\mathrm{avg}}$ reached during repetitive trials, and the logarithmic right-axis represents the corresponding number of input states *N* needed, while the red rectangular denotes the input state in our PQC, which is at least two orders of magnitude fewer than that in SQPT. It is noted that the data presented is valid for both fixed and variational weighting parameter conditions, since we utilized the same PQC configuration and achieved the same results. (**c**) VQPT on the weighted sum of the randomly generated quantum circuit on *n*-qubit, *d*-depth, and *N*-input configurations (denoted as (n,d,N)). The weighting probability p=0.1 is a fixed and known parameter. (**d**) VQPT on the weighted sum of the randomly generated quantum circuit on (6,7,35) cases with unknown weighting parameters *p*. We initialized the parameter $p_{\mathrm{init}}=0.1$, and we presented two trials with different target values $p_{\mathrm{opt}}=0.05$ and $p_{\mathrm{opt}}=0.20$ (red dotted lines). Results demonstrated convergence to the optimal values, and the maximum $F_{\mathrm{avg}}$ is over 99%.

A specific six-qubit randomly generated circuit is organized as shown in Figure 4a. Here, we first considered the weighted summation as a fixed and known structure to validate the capability of our VQPT method, where the weighting parameter is set at p=0.1. Hence, we utilized the ensemble of identity process *I* and the PQC $C(\vec{\theta })$ to approximate the non-unitary operator

(10) $$\begin{array}{c} C^{\prime }\left(\vec{\theta }\right)=0.1I+0.9C\left(\vec{\theta }\right). \end{array}$$

Numerically, we conducted the scalability tests up to eight-qubit cases, as shown in Figure 4b (Data details are listed in Table A1 in Appendix A). Results show a perfect reconstruction of the weighted summation operator with $F_{\mathrm{avg}}>99.99\%$. Meanwhile, compared to the number of input states needed in SQPT (dashed line), our method has at least two orders of magnitude fewer demands on the input states (rectangle marks). In Figure 4c, we give the variational optimization details of (4,5,8), (5,6,12) and (6,7,35) configurations, where we denote (n,d,N) as an *n*-qubit, *d*-depth, and *N*-input PQC configuration in numerical simulation. The cost function $f(\vec{\theta })$ and the fidelity $F_{\mathrm{avg}}$ both converge to the optimums.

- Variational weighted sum of PQCs

Further, we studied the case where the weighting parameter *p* is unknown,

(11) $$\begin{array}{c} C^{\prime }\left(\vec{\theta },p\right)=pI+\left(1-p\right)C\left(\vec{\theta }\right). \end{array}$$

As shown in Section 3.1, when conducting tomography on a non-unitary operator with an unknown weighting parameter, we could expand the specific cost function form with both circuit parameters $\vec{\theta }$ and weighting parameter *p*. The first item $\langle \psi_{j}^{\mathrm{ideal}}|\psi_{j}^{\mathrm{ideal}}\rangle$ in Equation (9) is a fixed value, and the last two items involve these parameters and can be further expanded with Equation (11):

(12) $$\begin{array}{cc} \langle \psi_{j}^{\mathrm{out}}|\psi_{j}^{\mathrm{out}}\rangle & =\langle \psi_{j}\left|{C^{\prime }\left(\vec{\theta },p\right)}^{†}C^{\prime }\left(\vec{\theta },p\right)\right|\psi_{j}\rangle \\ \; & =p^{2}\langle \psi_{j}|\psi_{j}\rangle +{\left(1-p\right)}^{2}\langle \psi_{j}\left|C{\left(\vec{\theta }\right)}^{†}C\left(\vec{\theta }\right)\right|\psi_{j}\rangle +2p\left(1-p\right)\langle \psi_{j}\left|C\left(\vec{\theta }\right)\right|\psi_{j}\rangle , \end{array}$$

(13) $$\begin{array}{cc} \langle \psi_{j}^{\mathrm{ideal}}|\psi_{j}^{\mathrm{out}}\rangle & =\langle \psi_{j}^{\mathrm{ideal}}\left|C^{\prime }\left(\vec{\theta },p\right)\right|\psi_{j}\rangle \\ \; & =p\langle \psi_{j}^{\mathrm{ideal}}|\psi_{j}\rangle +(1-p)\langle \psi_{j}^{\mathrm{ideal}}\left|C\left(\vec{\theta }\right)\right|\psi_{j}\rangle . \end{array}$$

Based on the expanded cost function items in Equations (12) and (13), we can calculate the corresponding gradient and conduct consequent optimization, where both $\vec{\theta }$ and *p* are variational targets. We give two examples of (6,7,35) with different weighting parameters $p_{\mathrm{opt}}=0.05$ and $p_{\mathrm{opt}}=0.20$, respectively, where the initial value is set as $p_{\mathrm{init}}=0.1$. Numerical results in Figure 4d show that our method is capable of optimizing the variational circuit parameters $\vec{\theta }$ (solid lines) together with the weighting parameter *p* (triangle and rectangular marks) based on the flexible cost function $f(\vec{\theta },p)$. Moreover, we conducted the scalability tests on unknown weighting parameter *p* cases, which achieved $F_{\mathrm{avg}}>99.99\%$ on all conditions as listed in Table A1 in Appendix A.

**Figure 5:**
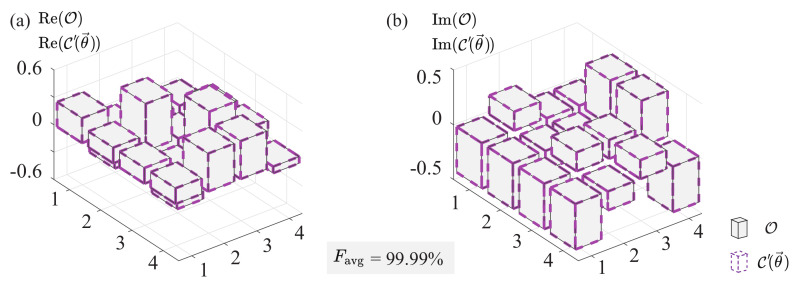
Density matrices of a two-qubit weighted sum of RQC. Gray shaded boxes are the target matrix and dashed purple boxes are the rebuilt $C^{\prime }\left(\vec{\theta }\right)$. Panels (**a**,**b**) are the corresponding real matrix and the imaginary matrix, respectively.

Here, we give a two-qubit case with the target matrix and the reconstructed matrix shown in Figure 5.

## 4. Case Study II: Imaginary Time Evolution of Heisenberg *XXZ* Spin Chain

We take the Hamiltonian of the Heisenberg *XXZ* spin chain [38] in a magnetic field as our example, which is written as,

(14) $${\hat{H}}_{\mathrm{XXZ}}=\sum_{l=1}^{n-1}\left[J\left({\hat{\sigma }}_{l}^{x}{\hat{\sigma }}_{l+1}^{x}+{\hat{\sigma }}_{l}^{y}{\hat{\sigma }}_{l+1}^{y}\right)+\Delta {\hat{\sigma }}_{l}^{z}{\hat{\sigma }}_{l+1}^{z}\right]+h\sum_{l=1}^{n}{\hat{\sigma }}_{l}^{z}.$$

Here *n* is the number of spins (qubits), *J* is the tunneling strength, Δ is the interaction strength, and *h* is the magnetization strength. The imaginary time evolutionary operator with time τ [39] is denoted as

(15) $$\begin{array}{c} O_{\mathrm{XXZ}}=e^{-{\hat{H}}_{\mathrm{XXZ}}\tau }. \end{array}$$

In the simulations, we fixed h=0.1 and J=1.

### 4.1. Methods

**Figure 6:**
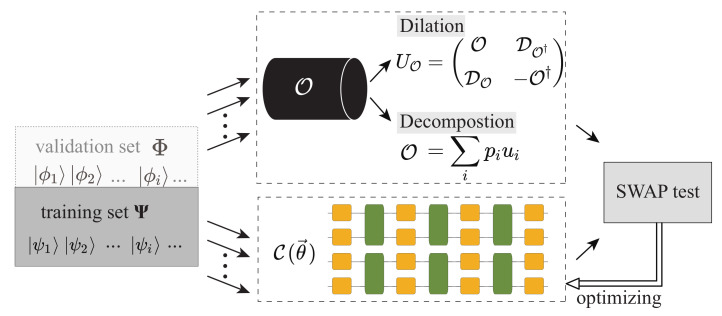
Mathematical transformation for non-unitary quantum process tomography, including dilation and decomposition.

Mathematically, we could transform the target non-unitary processes into unitary ones and afterward utilize the method in Ref. [26] to conduct consequent unitary learning, as shown in Figure 6. Here, we mainly introduce the following two methods.

- Unitary dilation

Every contraction operator on a small Hilbert space has a unitary dilation in an extended Hilbert space, which is guaranteed by the Sz.-Nagy dilation theorem [40].

**Theorem** **1**. *A contraction operator T applied up to N times on a smaller space H can be equivalent to a unitary $U_{T}$ applied up to N times on a larger space K, with*

(16) $$\begin{array}{c} T^{n}=P_{H}U_{T}^{n}P_{H},\;n\leq N, \end{array}$$

*where $P_{H}$ is the projection operator into space H.*

In a particular case, the Sz.-Nagy theorem has a minimal dilation when the extended space *K* has the smallest dimension. Here, we give an example of a minimal unitary dilation with N=1:

(17) $$\begin{array}{c} U_{T}=\left[\begin{array}{cc} T & D_{T^{†}}\\ D_{T} & -T^{†} \end{array}\right], \end{array}$$

where $D_{T}={\left(I-T^{†}T\right)}^{\frac{1}{2}}$.

Therefore, we can utilize Equation (17) to construct the extended unitary of the original non-unitary operator by adding one ancilla qubit.

- Unitary decomposition

Reference [41] shows that any quantum operator can be exactly decomposed as a linear combination of at most four unitary operators.

Specifically, any operator O can be decomposed into a Hermitian and an anti-Hermitian component:

(18) $$\begin{array}{cc} S & =\frac{1}{2}\left(O+O^{†}\right),\\ A & =\frac{1}{2}\left(O-O^{†}\right), \end{array}$$

such that O=S+A. Further, the Hermitian and anti-Hermitian components can be expressed as the sum of two unitary operators $u_{i}$:

(19) $$\begin{array}{cc} S & =\lim_{\epsilon \to 0}\frac{i}{2\epsilon }\left(e^{-i\epsilon S}-e^{i\epsilon S}\right)=\lim_{\epsilon \to 0}\frac{i}{2\epsilon }\left(u_{1}-u_{2}\right),\\ A & =\lim_{\epsilon \to 0}\frac{1}{2\epsilon }\left(e^{\epsilon A}-e^{-\epsilon A}\right)=\lim_{\epsilon \to 0}\frac{i}{2\epsilon }\left(u_{3}-u_{4}\right), \end{array}$$

as long as the expansion parameter ϵ approaches zero. With this decomposition, we can write the action of O as a sum of at most four unitary operators $u_{i}$, regardless of whether O itself is unitary or not.

### 4.2. Numerical Results

- Unitary dilation

Utilizing the unitary dilation method in Equation (17), we can dilate the non-unitary operator into a unitary by adding one ancilla qubit with the minimum dilation. Then, the problem is a trivial unitary one. Firstly, we numerically tested the five-qubit *XXZ* spin chain evolution with imaginary time τ varies from 0.01 to 0.15. By adding one ancilla qubit, we utilized the (6,7,20) PQC configuration to conduct the tomography. Table 1 shows a satisfying reconstruction of the extended unitary process with high $F_{\mathrm{avg}}$ and low $f(\vec{\theta })$.

Further, we fixed the evolution time τ=0.01, and conducted numerical simulations from four-qubit to six-qubit *XXZ* spin chain imaginary time evolution (the corresponding required qubits $n_{\mathrm{ext}}$ are extended to five to seven). It can be observed in Table 2 that with a shallow depth PQC and fewer input states, our method achieves satisfying results $F_{\mathrm{avg}}>99\%$. Similarly, in Section 3.2, the number of input states in our PQC $N_{\mathrm{PQC}}$ is far fewer than that in SQPT $N_{\mathrm{SQPT}}$.

- Unitary decomposition

As shown in Equation (19), we mathematically transformed the non-unitary quantum process tomography into four unitary quantum process tomography utilizing the unitary decomposition method and numerically simulated the cases up to four-qubit. Specifically, we prepared four unitary PQCs $C_{i}\left({\vec{\theta }}_{i}\right),i=1,2,3,4$ and superpose the four PQCs as shown in Equation (19) to approximate the non-unitary $O_{\mathrm{XXZ}}$. Therefore, the original non-unitary tomography is transformed into a unitary tomography with fourfold variational parameters. Taking two-qubit imaginary time evolution as an example, we numerically reconstructed the non-unitary quantum process O as the following four unitaries $C_{i}\left({\vec{\theta }}_{i}\right)$

Here, we choose the ϵ=0.05, and by Equations (18) and (19), we can obtain the superposition $C\left(\vec{\theta }\right)=10C_{1}\left({\vec{\theta }}_{1}\right)-10C_{2}\left({\vec{\theta }}_{2}\right)+10iC_{3}\left({\vec{\theta }}_{3}\right)-10iC_{4}\left({\vec{\theta }}_{4}\right)$ with average gate fidelity 99.90%. More details are presented in Table 2. It is worth noting from Table 2 and Table A1 that the accuracy value is a valid leading indicator to pick out the better PQC configurations with higher fidelity $F_{\mathrm{avg}}$ values. We give detailed data in Appendix B.

(20) $$\begin{array}{c} \begin{array}{c} C_{1}\left({\vec{\theta }}_{1}\right)=\left[\begin{array}{cccc} -0.004-0.781i & -0.245+0.078i & 0.265+0.170i & -0.258-0.395i\\ -0.199-0.080i & 0.332-0.509i & -0.103-0.535i & 0.116-0.522i\\ -0.403+0.279i & 0.037-0.354i & 0.364+0.650i & 0.163-0.222i\\ 0.087-0.306i & -0.036-0.659i & 0.209-0.057i & -0.044+0.642i \end{array}\right],\;\\ \;C_{2}\left({\vec{\theta }}_{2}\right)=\left[\begin{array}{cccc} 0.913-0.076i & -0.074-0.191i & 0.273+0.091i & -0.023+0.182i\\ -0.123+0.061i & -0.857-0.382i & -0.036+0.236i & -0.091-0.181i\\ -0.207-0.172i & 0.102-0.181i & 0.163+0.061i & -0.813+0.439i\\ 0.251-0.064i & -0.121+0.131i & -0.900-0.138i & -0.180+0.196i \end{array}\right],\\ \;C_{3}\left({\vec{\theta }}_{3}\right)=\left[\begin{array}{cccc} 0.625-0.377i & -0.159-0.554i & 0.321-0.025i & 0.071-0.156i\\ 0.302-0.422i & 0.511+0.575i & 0.259-0.165i & -0.058+0.198i\\ 0.096-0.349i & -0.129+0.010i & -0.415+0.562i & 0.308+0.517i\\ -0.072-0.245i & 0.235-0.049i & -0.141+0.540i & -0.620-0.423i \end{array}\right],\\ C_{4}\left({\vec{\theta }}_{4}\right)=\left[\begin{array}{cccc} -0.078+0.642i & 0.109-0.382i & 0.401-0.018i & -0.505+0.077i\\ 0.162-0.348i & 0.386-0.515i & -0.515-0.102i & -0.401-0.010i\\ 0.549-0.154i & -0.302+0.072i & 0.172+0.460i & -0.353-0.460i\\ -0.318-0.084i & -0.557-0.137i & -0.064-0.562i & -0.176-0.457i \end{array}\right]. \end{array} \end{array}$$

## 5. Conclusions

In this work, we propose a non-unitary variational quantum process tomography method based on the quantum machine learning algorithm, which encodes the unknown non-unitary quantum process into a PQC of a certain depth *d*. A set of randomly generated quantum states are used as the training data to minimize the cost function, and a validation set filters out the instance with the highest $F_{\mathrm{avg}}$ as a leading indicator, namely the closest parameter configuration to the unknown quantum process.

We introduce two methods to tackle the non-unitary quantum process tomography. One focuses on the mathematical transformation of the non-unitary problem, while the other attempts to utilize superpositions of PQCs to approximate the non-unitary.

We demonstrated our method by two numerical examples, including the superposition of the random quantum circuits and the imaginary time evolution with the Heisenberg *XXZ* spin chain from two-qubit to eight-qubit. The results indicate that a faithful reconstruction of O ($F_{\mathrm{avg}}$ higher than 99%) can be reached with a relatively low-depth PQC (d≤8), and a relatively small number of training states (at least two orders of magnitude compared to SQPT). Moreover, only a single qubit measurement is required in each configuration instead of measuring in the complete set of computational basis. Our work further proves the potential of the variational quantum process tomography framework on non-unitaries and presents a promising application of using the quantum machine learning algorithm to accelerate the quantum process tomography.

## Figures and Tables

**Table 1:** Numerical simulation results of the imaginary time evolution of the Heisenberg *XXZ* spin chain with varying time τ on (6,7,20) configuration utilizing unitary dilation method.

τ	Max. $F_{\mathrm{avg}}$	Avg. $f(\vec{\theta })$	τ	Max. $F_{\mathrm{avg}}$	Avg. $f(\vec{\theta })$
0.01	99.12%	0.061	0.09	94.54%	0.070
0.02	98.22%	0.062	0.10	94.07%	0.077
0.03	97.55%	0.039	0.11	93.76%	0.064
0.04	96.95%	0.061	0.12	93.44%	0.094
0.05	96.32%	0.051	0.13	93.22%	0.101
0.06	95.74%	0.070	0.14	93.03%	0.082
0.07	95.23%	0.074	0.15	92.99%	0.076
0.08	94.82%	0.053			

**Table 2:** Numerical simulation details of the imaginary time evolution of the Heisenberg *XXZ* spin chain from two-qubit to six-qubit cases utilizing unitary dilation or decomposition.

	*n*	$n_{\mathrm{ext}}$ ^1^	*d*	$N_{\mathrm{VQPT}}$ ^2^	$N_{\mathrm{SQPT}}$ ^3^	# of Paras. ^4^	Max. $F_{\mathrm{avg}}$	Max. Accuracy
Decomposition	2	−	2	4	16	72	99.90%	99.99%
	3	−	4	8	4096	180	99.61%	99.92%
	4	−	4	10	16,384	240	99.38%	99.89%
Dilation	4	5	6	10	1024	105	99.20%	99.61%
	5	6	7	20	4096	144	99.11%	99.34%
	6	7	8	55	16,384	189	99.23%	99.52%

^1^ Number of qubits in the extended space. Only dilation method needs one more qubit. ^2^ Number of input states in VQPT. ^3^ Number of input states in SQPT. ^4^ Number of parameters in $C\left(\vec{\theta }\right)$.

**Figure A1:**
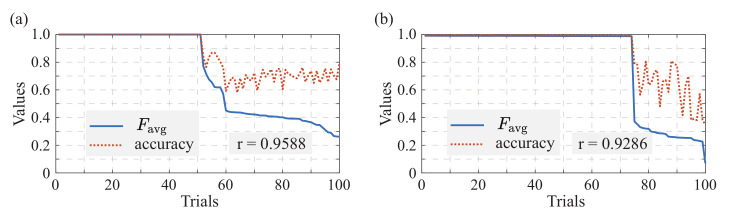
Correlations between the average gate fidelity $F_{\mathrm{avg}}$ and the accuracy. It is sorted as the $F_{\mathrm{avg}}$ value. Panels (**a**,**b**) give the 100 independent trials on the superposition of RQC and the imaginary time evolution of the Heisenberg *XXZ* Hamiltonian, respectively. Both cases show high Pearson correlation coefficient.

## Data Availability

The data presented in this study are available in the Tables in main text and Appendices Appendix A and Appendix B.

## Abbreviations

The following abbreviations are used in this manuscript:

VQPT	Variational quantum process tomography
SQPT	Standard quantum process tomography
PQC	Parametric quantum circuit
VQA	Variational quantum algorithm
RQC	Randomly-generated quantum circuit

## Appendix A

Here, we give the data details of the scalability tests corresponding to the Figure 4a content.

**Table A1:** Numerical simulation details of scalability test on randomly generated quantum circuits with d=2. $N_{\mathrm{PQC}}$ denotes the input states number in our variational method, and $N_{\mathrm{SQPT}}$ denotes the input states number in SQPT.

*n*	*d*	$N_{\mathrm{PQC}}$	$N_{\mathrm{SQPT}}$	# of Paras.	Max. $F_{\mathrm{avg}}$	Max. Accuracy
2	3	3	16	24	99.99%	99.99%
3	4	6	64	45	99.99%	99.99%
4	5	8	256	72	99.99%	99.99%
5	6	12	1024	105	99.99%	99.99%
6	7	35	4096	144	99.99%	99.99%
7	8	55	16,384	189	99.99%	99.99%
8	8	120	65,536	216	99.99%	99.99%

## Appendix B

Here, we give detailed data on the analysis of the two evaluation criteria: the average gate fidelity $F_{\mathrm{avg}}$ and the accuracy.

As stated in the main text, these two evaluation criteria are strongly correlated. Here, we conduct 100 independent trials and computed the two values. We utilize the Pearson’s correlation coefficient *r* [42] to evaluate such relations in our numerical simulations. The coefficient *r* ranges from −1 to 1, where r≥0.7 means highly linear positive correlated.

It is shown in Figure A1 that the accuracy value on the validation set has a strong correspondence with the final $F_{\mathrm{avg}}$ value with the Pearson correlation coefficient r=0.9588 and r=0.9286 on two typical non-unitary trials, respectively. Therefore, it is feasible to utilize the accuracy value as a criterion to determine the optimal circuit parameters to reconstruct O with higher $F_{\mathrm{avg}}$.
